# The Hospital for Sick Children

**Published:** 1915-12-11

**Authors:** Arthur Lucas, John Murray

**Affiliations:** Chairman. Great Ormond Street, London, W.C.; Vice-Chairman. Great Ormond Street, London, W.C.


*To the Editor of* The Hospital.

### The Hospital for Sick Children.

*To the Editor of* The Hospital.

Sir,—At this critical time, with ever-lengthening casualty lists and a falling birth-rate, the lives and health of the next generation become of paramount importance as a national asset; yet the urgent needs of our sick children are being overlooked. The Hospital for Sick Children, Great Ormond Street, where thousands of little ones have been restored to health and strength, is in serious straits. The ordinary sources of relief are dried up; and we can but appeal to the public in the belief that the work of the Hospital for Sick Children is so well known as to justify us in doing so.

Those hospitals which have been able to devote a part of their accommodation to the nursing of wounded soldiers are being paid for the work, and are able to keep their heads above water.

The Committee for the Hospital for Sick Children laid their plans for taking cases of this kind, but the needs of the children were so pressing (especially as children's wards have been converted into wards for soldiers in adult hospitals) that the scheme had to be abandoned, with the approval of the military authorities.

The Hospital for Sick Children is already in debt to its bankers for £5,400, and a further loan will probably have to be raised before Christmas. Surely such an institution as this ought not to be allowed to plead in vain for assistance at such a crisis.—Yours faithfully,

Arthur Lucas, Chairman.
John Murray, Vice-Chairman.

Great Ormond Street, London, W.C.,
December 3, 1915.

